# Exploring the diagnostic value of CLR and CPR in differentiating Kawasaki disease from other infectious diseases based on clinical predictive modeling

**DOI:** 10.3389/fped.2024.1345141

**Published:** 2024-02-16

**Authors:** Jin-Wen Liao, Xin Guo, Xu-Xia Li, Jia-Ming Xian, Cheng Chen, Ming-Guo Xu

**Affiliations:** ^1^ The Department of Pediatrics, Longgang District Maternity & Child Healthcare Hospital of Shenzhen City (Longgang Maternity and Child Institute of Shantou University Medical College), Shenzhen, Guangdong Province, China; ^2^ Neonatology, Longgang District Maternity & Child Healthcare Hospital of Shenzhen City (Longgang Maternity and Child Institute of Shantou University Medical College), Shenzhen, Guangdong Province, China; ^3^ The Department of Pediatrics, Third People’s Hospital of Longgang District Shenzhen, Shenzhen, Guangdong Province, China

**Keywords:** C-reactive protein to lymphocyte ratio, C-reactive protein to procalcitonin ratio, Kawasaki disease, infectious diseases, predictive model

## Abstract

**Background:**

Kawasaki disease (KD) is an important cause of acquired heart disease in children and adolescents worldwide. KD and infectious diseases can be easily confused when the clinical presentation is inadequate or atypical, leading to misdiagnosis or underdiagnosis of KD. In turn, misdiagnosis or underdiagnosis of KD can lead to delayed use of intravenous immunoglobulin (IVIG), increasing the risk of drug resistance and coronary artery lesions (CAL).

**Objectives:**

The purpose of this study was to develop a predictive model for identifying KD and infectious diseases in children in the hope of helping pediatricians develop timely and accurate treatment plans.

**Methods:**

The data Patients diagnosed with KD from January 2018 to July 2022 in Shenzhen Longgang District Maternity & Child Healthcare Hospital, and children diagnosed with infectious diseases in the same period will be included in this study as controls. We collected demographic information, clinical presentation, and laboratory data on KD before receiving IVIG treatment. All statistical analyses were performed using R-4.2.1 (https://www.rproject.org/). Logistic regression and Least Absolute Shrinkage with Selection Operator (LASSO) regression analyses were used to build predictive models. Calibration curves and C-index were used to validate the accuracy of the prediction models.

**Results:**

A total of 1,377 children were enrolled in this study, 187 patients with KD were included in the KD group and 1,190 children with infectious diseases were included in the infected group. We identified 15 variables as independent risk factors for KD by LASSO analysis. Then by logistic regression we identified 7 variables for the construction of nomogram including white blood cell (WBC), Monocyte (MO), erythrocyte sedimentation rate (ESR), alanine transaminase (ALT), albumin (ALB), C-reactive protein to procalcitonin ratio (CPR) and C-reactive protein to lymphocyte ratio (CLR). The calibration curve and C-index of 0.969 (95% confidence interval: 0.960–0.978) validated the model accuracy.

**Conclusion:**

Our predictive model can be used to discriminate KD from infectious diseases. Using this predictive model, it may be possible to provide an early determination of the use of IVIG and the application of antibiotics as soon as possible.

## 1 Introduction

Kawasaki disease (KD) is a common vasculitis syndrome in childhood whose major complication is coronary artery lesions (CAL), which has a significant impact on the cardiovascular health of children and adolescents (1). KD is a major culprit in cardiovascular and cerebrovascular sequelae in children, adolescents and even in adulthood (2, 3). An epidemiologic survey in Shanghai from 2013 to 2017 showed KD incidence rates ranging from 68.8 to 107.3 per 100,000 children younger than 5 years of age (4). Japan has a relatively high prevalence, with surveys during 2018 showing a KD prevalence of 359 per 100,000 children aged 0–4 years (5). Europe has a relatively low incidence of KD, with an annual incidence of KD of approximately 10–15 per 100,000 children under 5 years of age (6). In Shanghai, China, 8.4% of KD patients receiving initial intravenous immunoglobulin (IVIG) develop drug resistance, and 9.1% of KD patients develop CAL (4). Also in Japan, 19.7% of KD patients who received initial IVIG therapy did not respond, 9.0% of KD patients were diagnosed with cardiac complications within 30 days of disease onset, and 2.6% of KD patients developed cardiac sequelae after the acute phase (5). The incidence of KD is increasing year by year, whereas the rate of CAL is decreasing, and these can be attributed to advances in the early diagnosis and initial treatment of KD.

However, KD is prevalent between the ages of 0 and 5 years, which is the same time when infectious diseases in children are prevalent. Early diagnosis of KD relies on specific clinical manifestations. However, when the clinical manifestations are inadequate or atypical, KD and infectious diseases can be easily confused with each other, leading to misdiagnosis or underdiagnosis of KD (7). Ultrasound findings of typical coronary artery abnormalities are also an important indicator for diagnosing KD, but CAL tends to be delayed, which makes diagnosing KD with ultrasound lagging behind, and the optimal window of time for initial IVIG therapy in KD is within 10 days of onset, with delayed treatment leading to increased risk of drug resistance and CALs (8, 9). A recent study in Turkey also showed that prolonged duration of IVIG treatment is the most important determinant of the occurrence of CAL (10). Therefore, in order to avoid underdiagnosis and misdiagnosis of KD, new diagnostic markers should be actively developed on the basis of clinical presentation and cardiac ultrasound. Although there is no single validated hematologic marker that can be used to differentiate KD from infectious diseases in children, a subset of studies have been devoted to exploring clinical models for the combined identification of KD from other diseases by laboratory markers and blood biomarkers, with results suggesting that these models have more robust diagnostic performance (7, 11). This reveals that clinical predictive models that combine multiple indicators may be an important way out to solve the dilemma of KD diagnosis.

In fact many factors have been identified as biomarkers of Kawasaki disease, such as inflammatory biomarkers such as white blood cells (WBC), C-reactive protein (CRP) and procalcitonin (PCT), immunomarkers, proteomic markers, and genomic markers, but the common problem with these markers is that they have low sensitivity or specificity, and are of limited value when used individually to discriminate between KD and infectious diseases in children (12). C-reactive protein to procalcitonin ratio (CPR) helps to differentiate bacterial inflammation from non-bacterial inflammation, and C-reactive protein to lymphocyte ratio (CLR) is strongly correlated with the degree of infectious or non-infectious inflammatory response (13–15). Whether CLR and CPR are risk factors for KD is not yet known, and CLR and CPR have not yet been included in clinical prediction models for KD diagnosis.

In order to better identify children with Kawasaki disease (KD) and infectious diseases, we aimed to develop a clinical prediction tool with good predictive performance and strong interpretability by collecting demographic information and laboratory data from children, using lasso regression for variable screening and logistic regression for modelling, with a view to enhancing the early identification of children with KD by paediatricians.The study protocol and presentation of results were informed by the TRIPOD report list (16).

## 2 Methods

### 2.1 Data collection and inclusion criteria

The data Patients diagnosed with KD from January 2018 to July 2022 in Shenzhen Longgang District Maternity & Child Healthcare Hospital, and children diagnosed with infectious diseases in the same period will be included in this study as controls. Clinical information and laboratory data were collected separately for comparison between the KD and infection groups, and laboratory data for all KD patients were collected during the acute phase of KD and prior to treatment with IVIG. All children with KD were diagnosed according to the diagnostic criteria of the 2017 American Heart Association (AHA) guidelines (9). According to the guideline diagnostic criteria, a diagnosis of classic KD is made if the following conditions are met: fever for at least 5 days and at least 4 of the 5 main clinical features: 1. Erythema and cracking of lips, strawberry tongue, and/or erythema of oral and pharyngeal mucosa; 2. Bilateral bulbar conjunctival injection without exudate; 3. Rash: maculopapular, diffuse erythroderma, or erythema multiforme-like; 4. Erythema and edema of the hands and feet in acute phase and/or periungual desquamation in subacute phase; 5. Cervical lymphadenopathy (≥1.5 cm diameter), usually unilateral. Incomplete Kawasaki Disease is based on the flowchart mentioned in the guidelines for assessing suspected incomplete Kawasaki Disease. Infants were excluded from the study if they met any of the following criteria: autoimmune disease, inherited metabolic disease; unexplained fever; abandonment of hospitalization or incomplete information. Eventually 20 patients with KD and 4,132 infected children were excluded, and then 187 patients with KD and 1,190 patients with infectious diseases were included.

The study was conducted in accordance with the Declaration of Helsinki (revised 2013) and approved by the Ethics Committee of Shenzhen Longgang District Maternity & Child Healthcare Hospital (protocol code IRB No. LGFYYXLLL-2022-025 in 2022.09.29), which also approved the waiver of informed consent as this was a retrospective study and therefore did not require informed consent consent.

### 2.2 Power analysis

Our study focuses on two key variables, CLR and CPR. Therefore, we calculated the sample size based on these two variables using a specific formula. We set the parameters to have a type I error of *α *= 0.05, a type II error of *β *= 0.1, a sample size ratio of K = 1:1, and a threshold of *Δ *= 0. This ultimately led to a maximum sample size of 54. It is clear that the samples we collected greatly exceeded this maximum sample size.

### 2.3 Variable selection

Based on clinical experience and previous clinical studies (11, 17), two researchers from the team collected clinical information and laboratory data from children with KD and infected children. Variables collected included gender, age, weight, clinical presentation, white blood cell (WBC), hemoglobin (HGB), Hematocrit (HCT), mean corpuscular volume (MCV), mean corpuscular hemoglobin (MCH), mean corpuscular hemoglobin concentration (MCHC), Platelet (PLT), Mean platelet volume (MPV), Platelet distribution width (PDW), Monocyte (MO), erythrocyte sedimentation rate (ESR), alanine transaminase (ALT), albumin (ALB), aspartate transaminase (AST), neutrophil-to-lymphocyte ratio (NLR), platelet-to-lymphocyte ratio (PLR), CLR and CPR. The study was retrospective and masked information such as patient name and hospitalization number when obtaining subject information from the medical record system to minimize selection bias in the study. The data collectors were full-time pediatricians who were familiar with the inclusion and exclusion criteria of the study subjects. Laboratory data are measured by the examiner according to uniform standards, while the examiner does not know in advance that the subject will be included in the study, which reduces information bias.

### 2.4 Statistical analysis

All statistical analyses were performed using R-4.2.1 (https://www.rproject.org/). In order to demonstrate the characteristics of the distribution of the clinical and laboratory data in the two groups, we have performed descriptive statistics and one-way analyses of the parameters in the two groups. We tested whether the quantitative data of the two groups conformed to a normal distribution. Quantitative data that conformed to normal distribution were described statistically as mean ± standard deviation and analysed for differences using the independent samples *t*-test, while non-normally distributed measurements were described statistically as median (interquartile range) and analysed for differences using the Mann–Whitney U rank sum test. Count data were statistically described as frequencies and percentages and analysed for differences using the chi-square test. The “glmnet” package in the R software was used to perform least absolute shrinkage and selection operator (LASSO) regressions to screen for the best predictors of KD (18). The predictors selected for the LASSO regression were included in the multivariate logistic regression analysis using the “rms” package to build the predictive model. Differences of *P* < 0.05 were considered statistically significant.

### 2.5 Predictive model and nomogram construction

We used LASSO regression analysis to determine the best predictors of KD, and the minimum coefficient λ was determined by cross-validation. The factors screened by the LASSO regression were incorporated into the logistic regression analysis to construct the predictive model. Then we presented the prediction model as a nomogram. The C-index was used to assess the predictive performance of the model, and the closer the C-index was to 1, the better the predictive ability. In addition, we use calibration curves and receiver operating characteristic curves (ROC) to show the performance of the model in terms of calibration and discrimination.

## 3 Results

### 3.1 Baseline clinical characteristics

A total of 1,377 children were enrolled in this study, 844 (61%) boys and 533 (59%) girls with a median age of 1.00 (1.00–2.00) years.187 patients with KD were included in the KD group and 1,190 children with infectious diseases were included in the infected group. Based on univariate analysis, we observed no statistical difference in age, MCH, MPV, PBW, MO between the two groups.WBC, MCV, PLT, ESR, ALT, NLR, PLR, CLR, CPR were significantly higher in KD group than in the infected group (*p* < 0.05), whereas the levels of HGB, HCT, MCHC, ALB, AST were significantly lower in KD group than in the infected group (*p* < 0.05). The general profile of the included children is shown in Table 1.

**Table 1:** Characteristics of the distribution of demographic information and laboratory data on children with KD and children with infectious diseases.

	Overall, *N* = 1,377^a^	Infection disease *N* = 1,190^a^	KD, *N* = 187^a^	*p*-value^b^
AGE(years-old)	1.00 (1.00, 2.00)	1.00 (1.00, 2.00)	1.00 (1.00, 2.00)	0.003
GENDER(male)	844 (61%)	723 (61%)	121 (65%)	0.303
WBC (×10^9^/l)	9.5 (6.8, 13.6)	8.9 (6.6, 12.5)	15.0 (10.8, 18.2)	<0.001
HGB(g/l)	117 (110, 123)	118 (112, 125)	110 (103, 116)	<0.001
HCT	35.60 (33.70, 37.50)	35.80 (34.00, 37.80)	33.60 (31.70, 35.55)	<0.001
MCV	80 (78, 83)	80 (78, 83)	81 (78, 84)	0.035
MCH	27.00 (25.60, 27.60)	27.00 (25.22, 27.58)	27.00 (25.75, 27.65)	0.787
MCHC	328 (321, 336)	329 (321, 336)	327 (319, 334)	0.028
PLT(×10^9^/l)	296 (228, 377)	281 (222, 363)	366 (306, 435)	<0.001
MPV	9.60 (9.10, 10.20)	9.60 (9.03, 10.10)	9.70 (9.20, 10.20)	0.205
PDW	9.90 (9.00, 11.00)	9.90 (9.00, 11.00)	9.90 (9.10, 11.10)	0.434
MO	0.66 (0.45, 0.94)	0.65 (0.45, 0.93)	0.69 (0.49, 0.98)	0.052
ESR(mm/h)	22 (12, 43)	20 (11, 33)	69 (48, 86)	<0.001
ALT(U/l)	17 (14, 24)	17 (13, 22)	34 (17, 86)	<0.001
ALB(g/l)	42.3 (39.3, 44.8)	42.8 (40.4, 45.3)	36.5 (33.0, 39.2)	<0.001
AST(U/l)	40 (32, 49)	41 (33, 49)	34 (26, 52)	<0.001
NLR	1.25 (0.67, 2.56)	1.12 (0.62, 2.18)	2.58 (1.53, 4.65)	<0.001
PLR	85 (60, 126)	82 (58, 122)	107 (79, 158)	<0.001
CLR	4 (1, 12)	3 (1, 9)	18 (10, 39)	<0.001
CPR	53 (12, 167)	45 (10, 153)	123 (42, 392)	<0.001

KD, Kawasaki disease; WBC, white blood cell count; HGB, hemoglobin; HCT, Hematocrit; MCV, mean corpuscular volume; MCH, mean corpuscular hemoglobin; MCHC, mean corpuscular hemoglobin concentration; PLT, platelet count; MPV, Mean platelet volume; PDW, Platelet distribution width; MO, Monocyte; ESR, erythrocyte sedimentation rate; ALT, alanine aminotransferase; ALB, albumin; AST, aspartate aminotransferase; NLR, neutrophil-to-lymphocyte ratio; PLR, platelet-to-lymphocyte ratio; CLR, C-reactive protein to lymphocyte ratio; CPR, C-reactive protein to procalcitonin ratio.

^a^ Median (IQR) for quantitative data, *n* (%) for qualitative data.

^b^ Mann–Whitney U rank sum test analysis of quantitative data; Pearson's Chi-squared test analysis of qualitative data.

### 3.2 Selection of predictors

**Figure 1:**
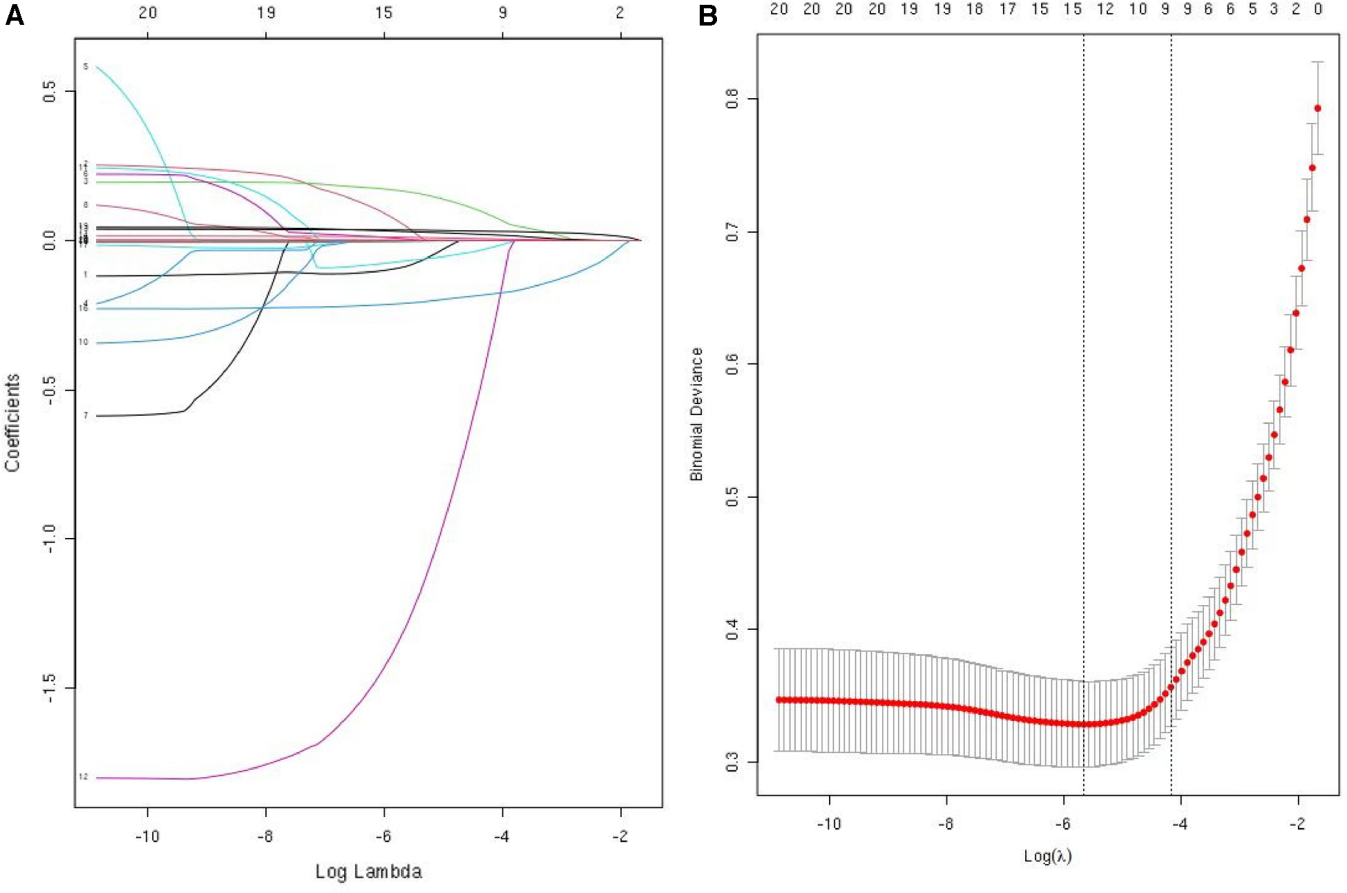
Use LASSO regression to select predictors. (**A**) LASSO regression was used to identify predictors. (**B**) Cross-validation and minimum criteria were used to adjust the penalty factor in the LASSO model. The vertical dashed line indicates the best lambda (the model that provides the best fit to the data).

**Figure 2:**
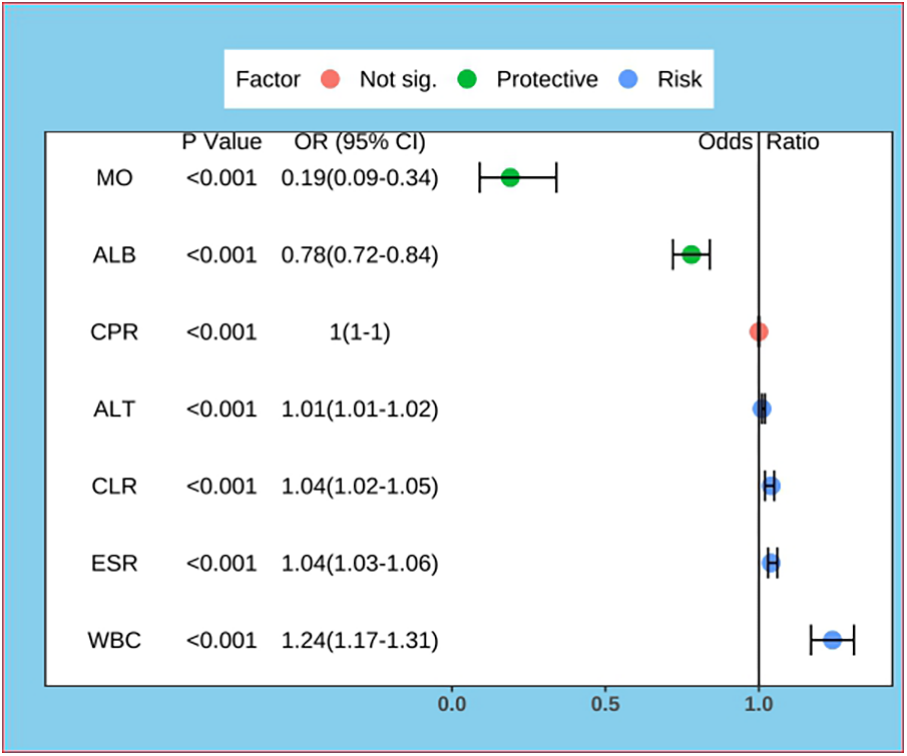
Forest plot for logistic regression of predictors. MO, Monocyte; ALB, albumin; CPR, C-reactive protein to procalcitonin ratio; ALT, alanine aminotransferase; CLR, C-reactive protein to lymphocyte ratio; ESR, erythrocyte sedimentation rate; WBC, white blood cell count.

The data were first downscaled using LASSO regression to screen for independent risk factors for KD (Figures 1A,B). When the coefficient *λ* is optimal, the coefficients *λ* of the excluded variables are compressed to zero, while the coefficients of the remaining variables in the model are not zero. The results of our analysis indicate that the optimal value of *λ* corresponds to 15 remaining variables (Figure 1B). Therefore, the results of LASSO analysis suggested that the 20 predictors were reduced to 15 predictors. Through multivariate logistic regression, we further filtered out 7 potential predictors with *P*-values < 0.05 in the logistic regression results from the above 15 predictors as the final variables for constructing the model (Figure 2). The seven predictors that were finalized included WBC, MO, ESR, ALT, ALB, CLR, and CPR.

### 3.3 Quantitative analysis of the degree of impact of CPR, CLR

**Figure 3:**
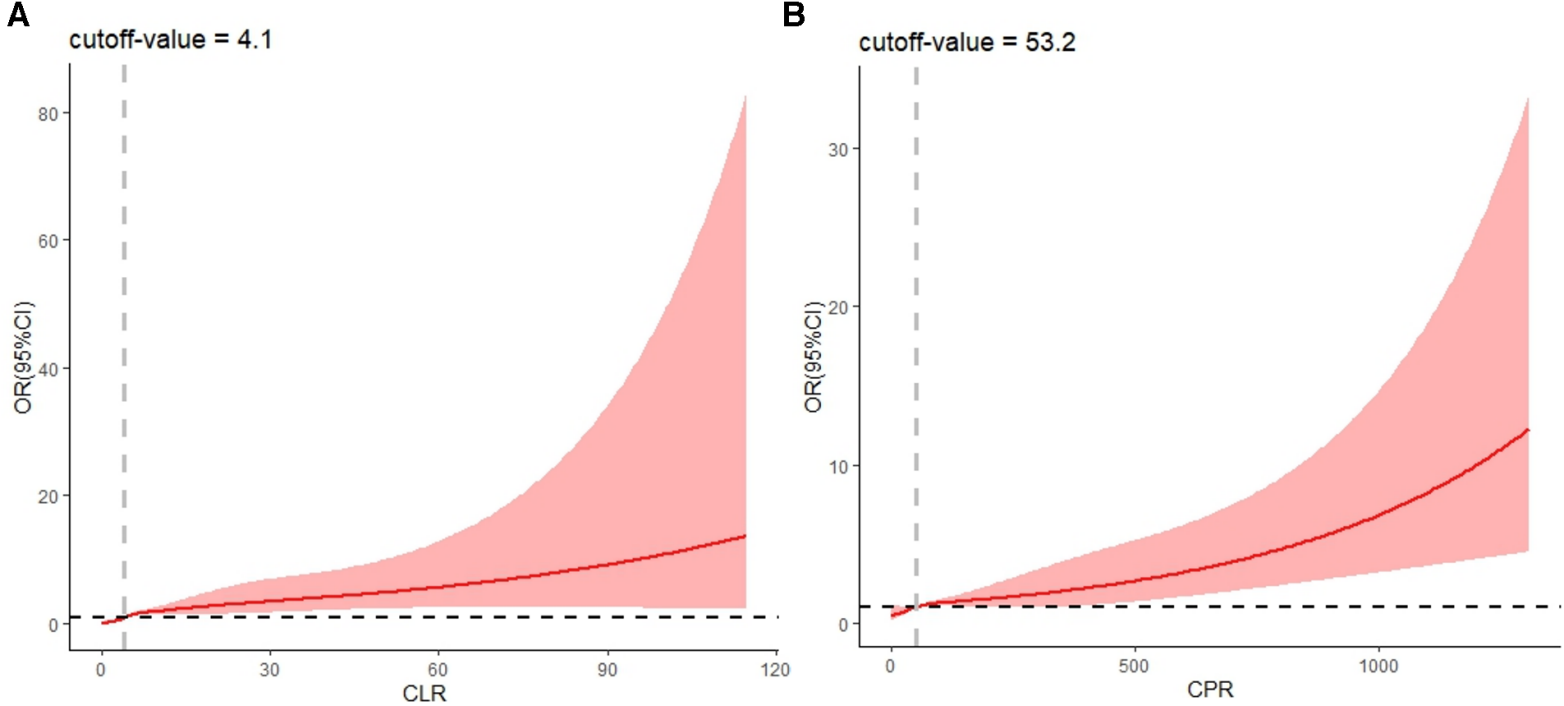
RCS plot of CLR and CPR (considering confounding effects of other predictors). CLR, C-reactive protein to lymphocyte ratio; CPR, C-reactive protein to procalcitonin ratio.

The effects of CPR and CLR on KD were analyzed by restricted cubic spline modeling after considering the effects exerted by other factors in the prediction model (19). The results suggested that when the value of CLR was greater than 4.1, the risk of KD gradually increased as the value of CLR gradually increased (Figure 3A). When the value of CPR was greater than 53.2, the risk of KD gradually increased as the value of CPR gradually increased (Figure 3B).

### 3.4 Development of nomogram

**Figure 4:**
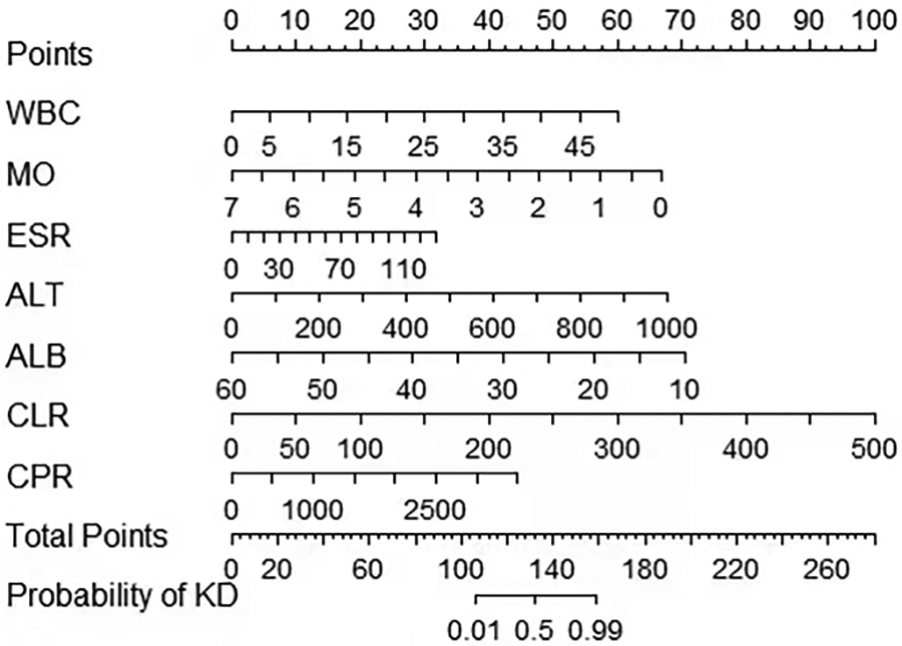
A nomogram to distinguish KD from infectious diseases in children. KD, Kawasaki disease; MO, Monocyte; ALB, albumin; CPR, C-reactive protein to procalcitonin ratio; ALT, alanine aminotransferase; CLR, C-reactive protein to lymphocyte ratio; ESR, erythrocyte sedimentation rate; WBC, white blood cell count.

A risk prediction nomogram was constructed from the seven variables with *P*-values < 0.05 in the logistic regression results (Figure 4). The C-index of the nomogram was 0.969 (95% confidence interval: 0.960–0.978), indicating that the model has good discriminatory and predictive ability.

### 3.5 Validation of the accuracy of the prediction model

**Figure 5:**
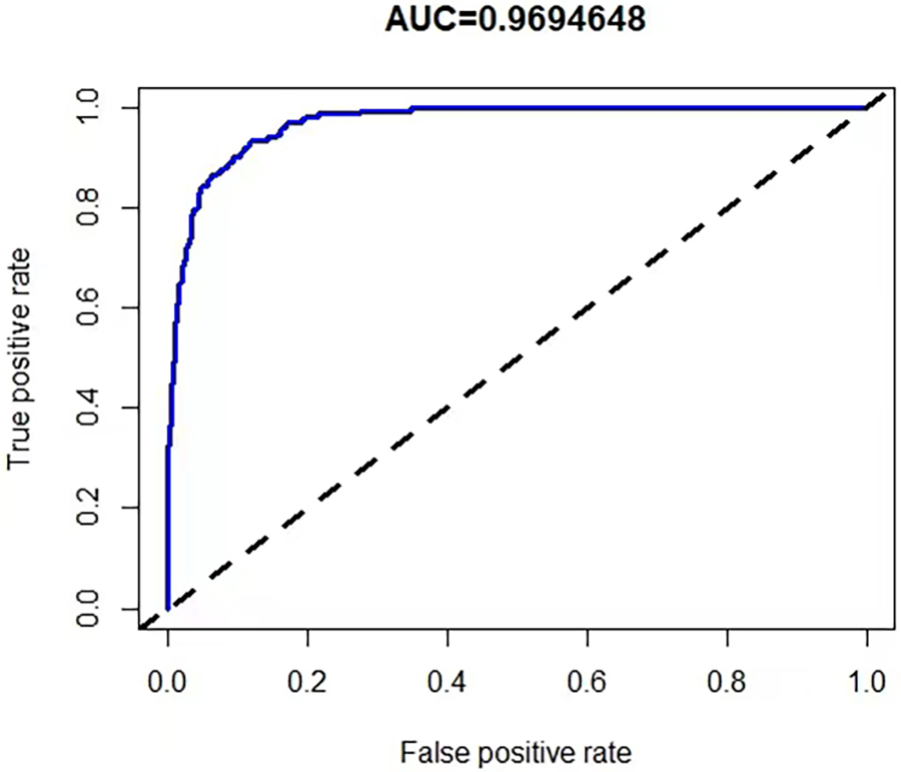
ROC curve for nomogram.

**Figure 6:**
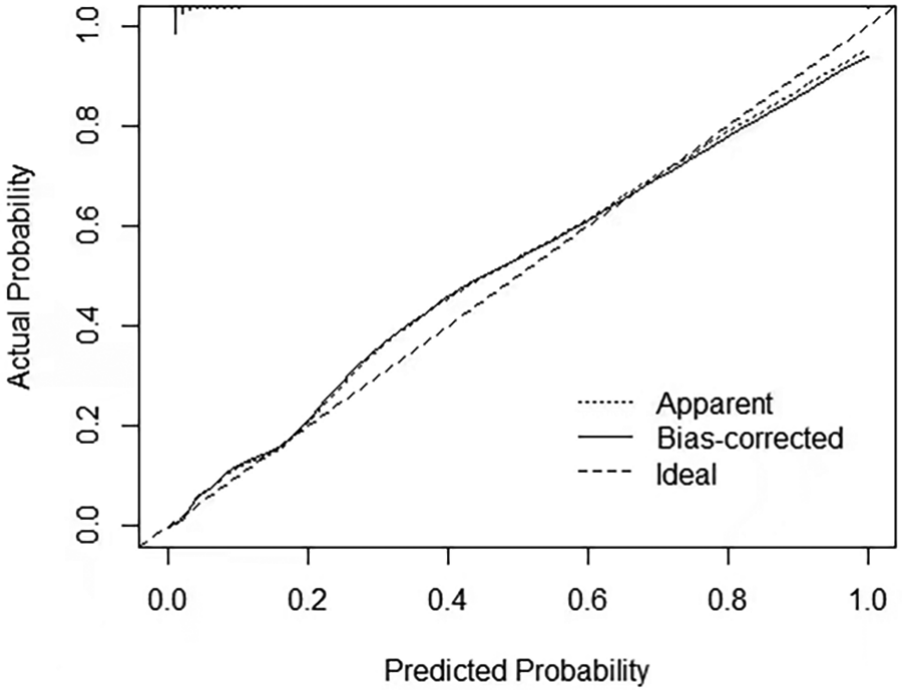
Calibration curve for nomogram.

To validate the accuracy of the predictive model, the ROC of the nomogram indicates that the model has high accuracy (Figure 5). In addition, calibration is required to plot the curves to evaluate the predictive model, and it can be seen that the predicted probabilities of the model remain essentially the same as the actual probabilities (Figure 6).

## 4 Discussion

In this study, we developed a predictive model for early recognition of Kawasaki disease and childhood infectious diseases. We found that WBC, MO, ESR, ALT, ALB, CLR and CPR were independent risk factors for KD. A nomogram constructed from these variables can be very helpful for pediatricians to identify KD and infectious diseases in children, thus reducing misdiagnosis and underdiagnosis of KD, as well as the incidence of cardiovascular sequelae. From our prediction model, we can see that WBC, ESR, ALT, CLR and CPR are risk factors for KD, and conversely ALB and MO are protective factors for KD.

Among the factors that differentiate KD from infectious diseases, the level of MO before IVIG treatment has the most significant effect. Disturbed immune response is one of the important pathogenetic mechanisms of KD, in which monocytes and their molecular markers can be used as diagnostic indicators of KD (20). Eight immune cells, including monocytes, construct a diagnostic scoring system that can be used to differentiate KD from febrile infections (21). All of the above studies suggested that monocytes are a risk factor for KD, but our study shows the opposite result. This may be related to the different subtypes of circulating monocytes, with the predominant classical CD14^++^CD16^−^ monocytes having phagocytosis of debris and tissue repair, whereas intermediate CD14^++^CD16^+^ monocytes lead to pro-inflammatory responses and reactive oxygen species generation (22). However, the role of monocytes in the pathogenesis and diagnosis of KD remains unclear and further studies are needed.

ALB is a traditional protective factor for KD and is a biomarker for predicting KD (23). ALB levels ≤3 g/dl can assist in determining incomplete KD, and low ALB levels have also been identified as an independent risk factor for CAL progression in patients with KD (9, 24).

From our results it is clear that CPR alone has little effect on the diagnosis of KD and being included in the model improves its predictive role for KD. Up to now no study has examined the relationship between CPR and KD. However, CPR has been found to have high predictive value in predicting infections, solid tumors, and ischemic stroke, which may be related to the property that PCT is elevated in infectious diseases more often than CRP (13, 25–27). Thus this index integrates the distinction between infectious and non-infectious inflammation, making it a biomarker that may differentiate KD from infectious disease.

Coincidentally, our study was the first to identify the role of CLR levels in the diagnosis of KD.CLR is a well-recognized indicator of inflammation with high predictive significance in both infectious and non-infectious inflammatory diseases (14, 15). CLR combines both inflammatory and immune factors in KD, and suggests that patients with KD have more intense inflammatory responses and immune disturbances compared to infected patients.

The ability of WBC, ESR and ALT alone to differentiate KD from infectious diseases is limited, so they need to be combined with other indicators for prediction (12). WBC and ALT can be used to differentiate KD from other febrile infections in children by constructing a nomogram in combination with other inflammatory indicators (23). ESR is highly expressed in KD and can be used as a diagnostic marker for KD (28).

Clinical predictive modeling, as a very effective tool, has a bright application prospect in KD diagnosis and prognosis. Liu et al. used eosinophil percentage, CRP, ALT, ALB, and WBC to construct a nomogram for predicting KD, and the AUC of the model was 0.873 (23). Huang et al. used total leukocyte count, prognostic nutritional index (PNI), complement C3, and NLR to construct a nomogram for differentiating KD from other febrile children, with AUCs of 0.858 and 0.825 for the training and validation groups, respectively (17). Liu et al. used WBC, HGB, PCT, CRP, ALT, and ALB to construct a predictive model for KD, with an AUC of 0.873 (29). Weng et al. then found that protein S100-A8, protein S100-A9, protein S100-A12, neutrophil defensin 1, alpha-1-acid glycoprotein 1 combined with six commonly used laboratory markers CRP, ALT, WBC, platelet, segment and hemoglobin can be used to differentiate KD from other febrile patients with a prediction model consisting of an AUC value of 0.88 (95% confidence interval: 0.80–0.96) (30). Tsai et al. found that combining eight independent predictors of platelets, eosinophil, alanine aminotransferase, CRP, hemoglobin, mean corpuscular hemoglobin, mean corpuscular hemoglobin concentration, and monocyte combined were used to distinguish KD from other febrile patients, and the sensitivity, specificity, and accuracy of the cohort were 0.824, 0.839, and 0.838, respectively (31). Our previous study incorporated WBC, hemoglobin, CRP, NLR, eosinophil-to-lymphocyte ratio, and PNI as indicators for constructing a KD prediction model with a C-index of 0.921 (11). In this study, we selected the indicators WBC, MO, ESR, ALT, ALB, CLR, and CPR to successfully construct a new KD prediction model with an AUC of 0.969, which achieved a new improvement in its predictive value over the previous models. In addition, compared with the above-mentioned studies, our study both used the 2017 AHA guidelines as the diagnostic criteria for KD. The current prediction models typically utilize a retrospective case-control study design, which provides some credibility to the conclusions drawn. However, when compared to prospective cohort study designs, retrospective case-control studies have limitations in controlling for bias and making causal inferences. Current prediction models commonly use children with infectious diseases as the control group, which is due to the clinical fact that KD is mainly distinguished from patients with infectious diseases.The current prediction models typically employ logistic regression for model construction.In terms of variable selection, Huang Y, GUO X, and Liu X utilize the traditional method of first considering individual variables and then aggregating them. This approach is feasible when dealing with fewer variables.When compared to their method of variable selection, the Lasso regression and support vector machines (SVM) utilized by ourselves and Weng K demonstrate superior adaptability to high-dimensional data, improved prediction performance, and greater interpretability.

Our established model still has some diagnostic limitations. Our study design is retrospective case-control, which has limitations in controlling for bias and making causal inferences. Therefore, the research conclusions should be validated in prospective cohort studies. Our model performs well in internal validation, but as we currently lack access to external data, external validation has not yet been conducted. Whether the model is overfitting still needs to be verified through multi-center cohort studies.Our model is primarily used to distinguish between children with Kawasaki disease and infectious diseases. It has not yet been applied to the differential diagnosis of Kawasaki disease and other diseases such as rheumatological autoimmune diseases. Therefore, there are certain limitations in the application scenarios of our model, and further improvements are needed.

## 5 Conclusion

Our study used common laboratory indicators WBC, MO, ESR, ALT, ALB, CPR, CLR to construct a prediction model of Kawasaki disease. By using the model, we can distinguish Kawasaki disease from other infectious diseases. Using this prediction model, it is helpful to the early diagnosis of Kawasaki disease and to start the use of IVIG as early as possible, thus reducing the incidence of CAL. It could also reduce the overuse of antibiotics.

## Data Availability

The raw data supporting the conclusions of this article will be made available by the authors, without undue reservation.
